# Crystal structure of octa­kis­(*N*,*N*-di­methyl­formamide-κ*O*)europium(III) tetra­cosa-μ_2_-oxido-dodeca­oxido-μ_12_-phosphato-dodeca­molybdate(VI)

**DOI:** 10.1107/S2056989016003546

**Published:** 2016-03-04

**Authors:** Yassine Ghandour, Imen Hammami, Shabir Najmudin, Cecilia Bonifácio, Mohamed Salah Belkhiria

**Affiliations:** a Laboratoire de Physico-chimie des Matériaux, Faculté des Sciences de Monastir, Avenue de l’environnement, 5019 Monastir, University of Monastir, Tunisia; b Faculdade de Medicina, Veterinària, Universidade Tecnica de Lisboa, Avenida da Universidade Tecnica, 1300-477 Lisboa, Portugal; c REQUIMTE/CQFB Departamento de Quimica, Faculdade de Ciencias e Tecnologia, Universidade Nova de Lisboa, 2829-516 Caparica, Portugal

**Keywords:** crystal structure, α-Keggin-type [PMo_12_O_40_]^3−^ polyanion, europium, IR spectroscopy

## Abstract

In the title salt, [Eu(C_3_H_7_NO)_8_][PMo_12_O_40_], the asymmetric unit comprises one α-Keggin-type [PMo_12_O_40_]^3−^ polyoxidometalate anion and one distorted dodeca­hedral [Eu(C_3_H_7_NO)_8_]^3+^ complex cation. In the crystal, the isolated polyoxidometalate anions are packed into hexa­gonally arranged rows extending parallel to [001]. The complex cations are situated between the rows and are linked to the neighbouring anions through weak C—H⋯O hydrogen-bonding inter­actions, leading to the formation of a three-dimensional network structure.

## Abstract 2

The asymmetric unit of the title compound consists of one [Eu(C_3_H_7_NO)_8_]^3+^ complex cation and one α-Keggin-type [PMo_12_O_40_] polyanion. Cations and anions are linked through C—H⋯O hydrogen bonds.

## Chemical context   

Polyoxidometalates (POMs) are versatile metal–oxygen complexes which have attracted inter­est due to their topolog­ical properties and their potential applications in catalysis, photoluminescence, electrochromism and magnetism (Long *et al.*, 2010 ▸; Pope & Müller, 2010 ▸; Coronado & Gómez-García, 1998 ▸). Up to date, a variety of strategies have been developed and used to assemble POM-based hybrid materials by controlling reaction factors such as metal ions, organic ligands, POM species, pH, molar ratio of raw materials or reaction environments (Wang *et al.*, 2013 ▸; Liu *et al.*, 2013 ▸). Even with these approaches, the design and synthesis of new stable polyoxidomolybdate complexes are still challenging.
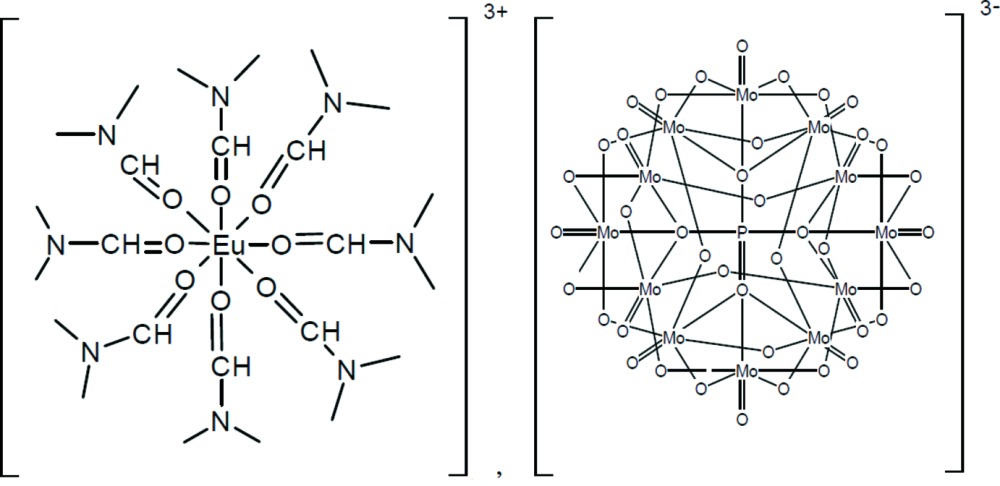


Herein, we report on the synthesis, UV–vis and IR spectra along with the crystal structure of the hybrid europium(III) POM title compound, [Eu(C_3_H_7_NO)_8_][PMo_12_O_40_], (I).

## Structural commentary   

**Figure 1:**
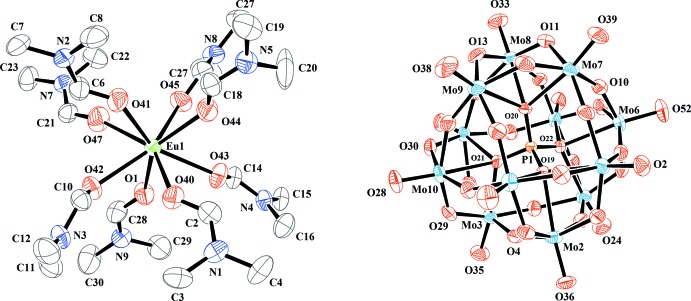
The mol­ecular structures of the cation and anion in compound (I), showing the atom-numbering scheme. Displacement ellipsoids are drawn at the 40% probability level. H atoms have been omitted for clarity.

The structures of the mol­ecular components of compound (I) are illustrated in Fig. 1 ▸. The [PMo_12_O_40_]^3−^ polyoxidoanion of (I) exhibits a classical α-Keggin-type structure. The central P atom is tetra­hedrally surrounded with all four oxygen atoms (O_a_) linked to four Mo_3_O_13_ moieties. The latter species are fused together by sharing corner atoms (O_b_) and consist of three MoO_6_ octa­hedra condensed in a triangular arrangement by sharing edges (O_c_). There is also a terminal oxygen atom (O_d_) in every MoO_6_ octa­hedron. The P—O bond lengths range from 1.521 (5) Å to 1.536 (4) Å and the Mo—O bond lengths from 1.690 (5) Å to 2.438 (4) Å. The O—P—O angles [109.1 (2)–109.8 (3)°] indicate only a slight distortion of the central PO_4_ tetra­hedron. The Eu^III^ cation is coordinated by eight di­methyl­formamide ligands through their oxygen atoms with Eu—O distances from 2.369 (5) to 2.416 (6) Å. These values are comparable to those of related oxido-europium(III) species, *e.g* for the [Eu(thd)_3_(DMF)_2_] complex (thd is the ion of 2,2,6,6-tetra­methyl-3,5-hepta­nedione) with Eu—O = 2.494 (5)–2.442 (5) Å (Cunningham & Siever, 1980 ▸). Calculations with the *SHAPE* software (Alvarez *et al.*, 2005 ▸) indicate that the coordination polyhedron of Eu^III^ is a slightly distorted dodeca­hedron approaching mol­ecular *D*_2*d*_ symmetry (Casanova *et al.*, 2005 ▸).

## Supra­molecular features   

**Figure 2:**
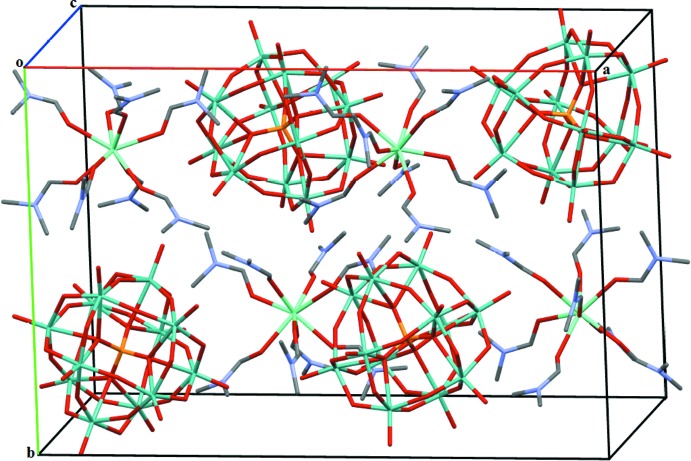
The contents of the unit cell of complex (I). H atoms have been omitted for clarity.

**Figure 3:**
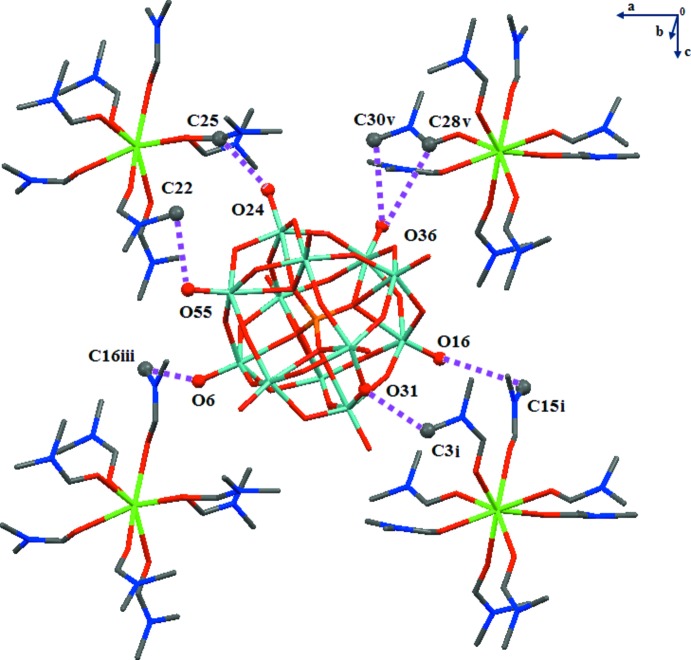
C—H⋯O hydrogen bonds (dashed lines) link one [Eu(dmf)_8_]^3+^ cation to four neighbouring α-Keggin-type [PMo_12_O_40_]^3−^ anions. Symmetry codes refer to Table 1 ▸.

**Figure 4:**
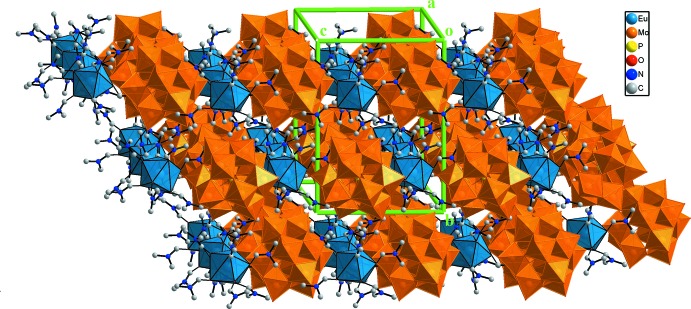
The crystal packing of (I) with the [PMo_12_O_40_]^3−^ anions in polyhedral representation.

The unit cell content of the title compound is illustrated in Fig. 2 ▸. In the crystal structure of (I), each [Eu(DMF)_8_]^3+^ cation is linked to four neighbouring α-Keggin-type [PMo_12_O_40_]^3−^ anions through C—H⋯O hydrogen-bonding inter­actions between the methyl groups of the DMF ligands and the terminal-oxygen (O_d_) and the bridging-oxygen atoms (O_b,c_) of the [PMo_12_O_40_]^3−^ anions (Fig. 3 ▸, Table 1 ▸). The C(donor)⋯O_d_(acceptor) distances are between 3.174 (10) and 3.541 (11) Å while the C⋯O(_b,c_) distances are between 3.289 (11) and 3.473 (12) Å. In the crystal packing, the POM anions are packed into hexa­gonally arranged rows extending parallel to [001] with the [Eu(DMF)_8_]^3+^ cations located between the rows (Fig. 4 ▸).

## Synthesis and crystallization   

The starting material [(C_4_H_9_)_4_N)_4_H_3_][PMo_11_O_39_] was prepared using a literature method (Combs-Walker & Hill, 1998 ▸). EuCl_3_·6H_2_O (361.41 mg, 1 mmol) and isonicotinic acid (C_6_H_5_NO_2_) (123.11 mg, 1 mmol) were dissolved in 10 ml of di­methyl­formamide. This solution was added dropwise to a yellow di­methyl­formamide solution of [(C_4_H_9_)_4_N)_4_H_3_][PMo_11_O_39_] (0.33 mmol in 10 ml). The mixture was heated under stirring for 1 h at 333 K. Single crystals of the title compounds were obtained by slow diffusion of 2-propanol through the di­methyl­formamide solution. UV–vis spectrum in di­methyl­formamide: λ_max_ (nm) 315 and 205.

## FT–IR spectroscopy   

The FT–IR spectrum was recorded in the range 4000–400 cm^−1^ on a Nicolet 470 FT–IR spectrophotometer with pressed KBr pellets.

**Figure 5:**
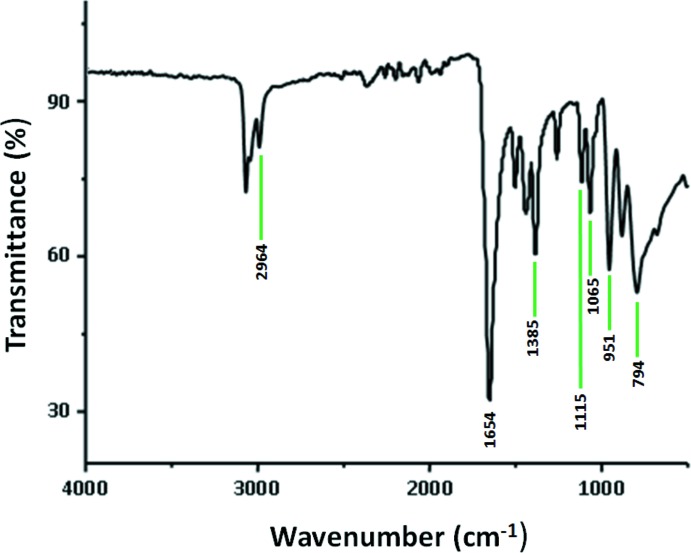
The FT–IR spectrum of (I).

The FT–IR spectrum of (I) (Fig. 5 ▸) exhibits characteristic bands attributed to the stretching and deformation modes of the Mo—O bond vibration of the [PMo_12_O_40_]^3−^ anion in the region 1100–400 cm^−1^_._ Thus, the asymmetric vibration ν_as_(P—O_a_), ν_as_(Mo=O_d_), ν_as_(Mo—O_b_—Mo) and ν_as_(Mo—O_c_—Mo) appear at 1065, 951, 885 and 974 cm^−1^, respectively (Masteri-Farahani & Shahbazi, 2012 ▸). The absorption bands at 1265 and 1657 cm^−1^ are characteristic of the asymmetric vibration of the C—N and the C=O bonds, respectively. The vibration bands at 1115, 1440, 1385 and 2964 cm^−1^ are attributed to the vibration ρ(CH_3_) (rocking vibration), δ_a_(CH_3_), δ_s_(CH_3_) and ν(C-H) of the di­methyl­formamide ligand (Durgaprasad *et al.*, 1971 ▸).

## Refinement   

Crystal data, data collection and structure refinement details are summarized in Table 2 ▸. Hydrogen atoms were placed in calculated positions and refined as riding atoms: C—H = 0.93 Å and *U*_iso_(H) = 1.2*U*_eq_(C) for methine groups and C—H = 0.96 Å and *U*_iso_(H) = 1.5*U*_eq_(C) for methyl groups. The refined Flack parameter (Parsons *et al.*, 2013 ▸) of −0.015 (7) indicates the correct determination of the absolute structure.

## Supplementary Material

Crystal structure: contains datablock(s) I. DOI: 10.1107/S2056989016003546/wm5274sup1.cif

Structure factors: contains datablock(s) I. DOI: 10.1107/S2056989016003546/wm5274Isup2.hkl

CCDC reference: 1456619

Additional supporting information:  crystallographic information; 3D view; checkCIF report

## Figures and Tables

**Table 1:** Hydrogen-bond geometry (Å, °)

*D*—H⋯*A*	*D*—H	H⋯*A*	*D*⋯*A*	*D*—H⋯*A*
C3—H4⋯O31^i^	0.96	2.72	3.289 (11)	118
C4—H7⋯O36^ii^	0.96	2.61	3.476 (12)	150
C7—H13⋯O7	0.96	2.65	3.473 (12)	145
C8—H15⋯O28^ii^	0.96	2.51	3.316 (13)	141
C8—H16⋯O5	0.96	2.58	3.424 (11)	147
C10—H18⋯O28^ii^	0.93	2.62	3.516 (10)	162
C15—H29⋯O16^i^	0.96	2.55	3.299 (12)	135
C15—H27⋯O32^iii^	0.96	2.52	3.440 (10)	160
C15—H28⋯O52^iv^	0.96	2.29	3.174 (10)	153
C16—H31⋯O6^iii^	0.96	2.63	3.541 (11)	159
C22—H41⋯O55	0.96	2.53	3.280 (12)	135
C22—H42⋯O11^iv^	0.96	2.45	3.305 (11)	149
C22—H43⋯O45	0.96	2.61	3.567 (13)	172
C23—H44⋯O17^v^	0.96	2.52	3.443 (11)	161
C25—H55⋯O24	0.93	2.53	3.337 (11)	145
C25—H55⋯O44	0.93	2.58	3.091 (10)	115
C28—H56⋯O36^v^	0.93	2.62	3.476 (11)	153
C30—H60⋯O36^v^	0.96	2.58	3.458 (14)	152

Symmetry codes: (i) 
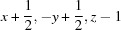
; (ii) 
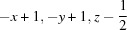
; (iii) 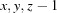; (iv) 
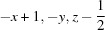
; (v) 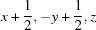.

**Table 2:** Experimental details

Crystal data	
Chemical formula	[Eu(C_3_H_7_NO)_8_][PMo_12_O_40_]
*M*_r_	2558.97
Crystal system, space group	Orthorhombic, *P* *n* *a*2_1_
Temperature (K)	296
*a*, *b*, *c* (Å)	26.9108 (10), 18.3506 (6), 13.4494 (4)
*V* (Å^3^)	6641.7 (4)
*Z*	4
Radiation type	Mo *K*α
μ (mm^−1^)	3.24
Crystal size (mm)	0.20 × 0.18 × 0.17

Data collection	
Diffractometer	Bruker APEXII CCD
Absorption correction	Multi-scan (*SADABS*; Bruker, 2006 ▸)
*T*_min_, *T*_max_	0.667, 0.747
No. of measured, independent and observed [*I* > 2σ(*I*)] reflections	88354, 33285, 19583
*R*_int_	0.075
(sin θ/λ)_max_ (Å^−1^)	0.849

Refinement	
*R*[*F* ^2^ > 2σ(*F* ^2^)], *wR*(*F* ^2^), *S*	0.047, 0.095, 0.98
No. of reflections	33285
No. of parameters	863
No. of restraints	1
H-atom treatment	H-atom parameters constrained
Δρ_max_, Δρ_min_ (e Å^−3^)	2.08, −1.80
Absolute structure	Flack *x* determined using 6924 quotients [(*I* ^+^)−(*I* ^−^)]/[(*I* ^+^)+(*I* ^−^)]
Absolute structure parameter	−0.015 (7)

Computer programs: *APEX2* and *SAINT* (Bruker, 2006 ▸), *SIR2004* (Burla *et al.*, 2005 ▸), *SHELXL2014* (Sheldrick, 2015 ▸), *ORTEP-3 for Windows* (Farrugia, 2012 ▸), *DIAMOND* (Putz & Brandenburg, 2014 ▸) and *WinGX* (Farrugia, 2012 ▸).

## supplementary crystallographic information

### Crystal data

**Table:**

[Eu(C_3_H_7_NO)_8_][PMo_12_O_40_]	*D*_x_ = 2.559 Mg m^−^^3^
*M**_r_* = 2558.97	Mo *K*α radiation, λ = 0.71073 Å
Orthorhombic, *P**n**a*2_1_	Cell parameters from 9528 reflections
*a* = 26.9108 (10) Å	θ = 3.0–32.3°
*b* = 18.3506 (6) Å	µ = 3.24 mm^−^^1^
*c* = 13.4494 (4) Å	*T* = 296 K
*V* = 6641.7 (4) Å^3^	Prism, yellow
*Z* = 4	0.20 × 0.18 × 0.17 mm
*F*(000) = 4888	

### Data collection

**Table:**

Bruker APEXII CCD diffractometer	19583 reflections with *I* > 2σ(*I*)
Radiation source: fine-focus sealed tube	*R*_int_ = 0.075
φ and ω scans	θ_max_ = 37.1°, θ_min_ = 2.2°
Absorption correction: multi-scan (*SADABS*; Bruker, 2006)	*h* = −41→45
*T*_min_ = 0.667, *T*_max_ = 0.747	*k* = −31→31
88354 measured reflections	*l* = −21→22
33285 independent reflections	

### Refinement

**Table:**

Refinement on *F*^2^	Secondary atom site location: difference Fourier map
Least-squares matrix: full	Hydrogen site location: inferred from neighbouring sites
*R*[*F*^2^ > 2σ(*F*^2^)] = 0.047	H-atom parameters constrained
*wR*(*F*^2^) = 0.095	*w* = 1/[σ^2^(*F*_o_^2^) + (0.035*P*)^2^] where *P* = (*F*_o_^2^ + 2*F*_c_^2^)/3
*S* = 0.98	(Δ/σ)_max_ = 0.001
33285 reflections	Δρ_max_ = 2.08 e Å^−^^3^
863 parameters	Δρ_min_ = −1.80 e Å^−^^3^
1 restraint	Absolute structure: Flack *x* determined using 6924 quotients [(*I*^+^)-(*I*^-^)]/[(*I*^+^)+(*I*^-^)]
Primary atom site location: structure-invariant direct methods	Absolute structure parameter: −0.015 (7)

### Special details

**Table:**

Geometry. All e.s.d.'s (except the e.s.d. in the dihedral angle between two l.s. planes) are estimated using the full covariance matrix. The cell e.s.d.'s are taken into account individually in the estimation of e.s.d.'s in distances, angles and torsion angles; correlations between e.s.d.'s in cell parameters are only used when they are defined by crystal symmetry. An approximate (isotropic) treatment of cell e.s.d.'s is used for estimating e.s.d.'s involving l.s. planes.
Refinement. Refinement of *F*^2^ against ALL reflections. The weighted *R*-factor *wR* and goodness of fit *S* are based on *F*^2^, conventional *R*-factors *R* are based on *F*, with *F* set to zero for negative *F*^2^. The threshold expression of *F*^2^ > 2σ(*F*^2^) is used only for calculating *R*-factors(gt) *etc*. and is not relevant to the choice of reflections for refinement. *R*-factors based on *F*^2^ are statistically about twice as large as those based on *F*, and *R*- factors based on ALL data will be even larger.

### Fractional atomic coordinates and isotropic or equivalent isotropic displacement parameters (Å^2^)

**Table:**

	*x*	*y*	*z*	*U*_iso_*/*U*_eq_	
Eu1	0.62226 (2)	0.24667 (2)	0.27301 (3)	0.03117 (7)	
Mo1	0.26680 (2)	0.18597 (3)	0.63650 (5)	0.03332 (13)	
Mo2	0.30169 (2)	0.35458 (3)	0.55755 (5)	0.03366 (13)	
Mo3	0.41829 (3)	0.43690 (3)	0.64996 (5)	0.03387 (13)	
Mo4	0.47559 (2)	0.36195 (3)	0.85122 (5)	0.03004 (12)	
Mo5	0.49335 (2)	0.20234 (3)	0.68682 (5)	0.03324 (13)	
Mo6	0.39245 (3)	0.10613 (3)	0.60967 (5)	0.03505 (14)	
Mo7	0.32991 (3)	0.10420 (3)	0.85410 (5)	0.03838 (15)	
Mo8	0.43839 (3)	0.17833 (4)	0.93808 (5)	0.03661 (14)	
Mo9	0.32843 (3)	0.26018 (4)	0.99282 (5)	0.04260 (17)	
Mo10	0.36044 (2)	0.43617 (3)	0.87490 (5)	0.03517 (14)	
Mo11	0.25072 (2)	0.33286 (4)	0.78760 (5)	0.03541 (14)	
Mo12	0.42166 (3)	0.26738 (4)	0.49994 (5)	0.03432 (13)	
P1	0.37299 (6)	0.26883 (8)	0.74508 (12)	0.0207 (3)	
O1	0.6764 (2)	0.1551 (4)	0.2093 (5)	0.0526 (16)	
O2	0.2228 (2)	0.1295 (3)	0.5946 (5)	0.0453 (14)	
O3	0.26515 (19)	0.2607 (3)	0.5440 (4)	0.0350 (11)	
O4	0.34964 (18)	0.4169 (3)	0.6033 (4)	0.0320 (10)	
O5	0.47195 (19)	0.4273 (3)	0.7347 (4)	0.0325 (11)	
O6	0.5310 (2)	0.3873 (3)	0.8969 (4)	0.0420 (13)	
O7	0.49746 (19)	0.2908 (3)	0.7483 (4)	0.0330 (11)	
O8	0.45951 (19)	0.1127 (2)	0.6394 (4)	0.0355 (11)	
O9	0.32412 (19)	0.1439 (3)	0.5897 (4)	0.0333 (11)	
O10	0.3722 (2)	0.0854 (3)	0.7370 (4)	0.0372 (12)	
O11	0.3952 (2)	0.1009 (3)	0.9240 (4)	0.0407 (13)	
O12	0.45956 (19)	0.2814 (3)	0.9252 (4)	0.0349 (11)	
O13	0.3888 (2)	0.2209 (3)	1.0310 (4)	0.0420 (13)	
O14	0.3515 (2)	0.3523 (3)	0.9652 (4)	0.0374 (12)	
O15	0.30057 (19)	0.4081 (3)	0.8207 (4)	0.0337 (11)	
O16	0.1992 (2)	0.3743 (4)	0.8289 (5)	0.0527 (16)	
O17	0.25496 (19)	0.3787 (3)	0.6547 (4)	0.0363 (11)	
O18	0.22650 (19)	0.2506 (3)	0.7233 (4)	0.0377 (12)	
O19	0.32137 (16)	0.2804 (2)	0.7018 (3)	0.0231 (9)	
O20	0.36948 (17)	0.2233 (2)	0.8397 (3)	0.0257 (9)	
O21	0.39628 (16)	0.3431 (2)	0.7695 (3)	0.0241 (8)	
O22	0.40539 (17)	0.2288 (2)	0.6696 (3)	0.0248 (9)	
O23	0.35674 (19)	0.2989 (3)	0.5004 (4)	0.0344 (11)	
O24	0.4401 (3)	0.2850 (4)	0.3828 (4)	0.0512 (15)	
O25	0.43539 (19)	0.3610 (3)	0.5671 (4)	0.0342 (11)	
O26	0.48598 (18)	0.2386 (3)	0.5597 (4)	0.0340 (11)	
O27	0.4063 (2)	0.1693 (3)	0.4917 (4)	0.0349 (11)	
O28	0.3447 (2)	0.5055 (3)	0.9514 (5)	0.0499 (15)	
O29	0.38277 (19)	0.4838 (2)	0.7624 (4)	0.0361 (11)	
O30	0.43123 (19)	0.4244 (3)	0.9129 (4)	0.0344 (11)	
O31	0.3075 (2)	0.1592 (3)	0.9607 (4)	0.0440 (14)	
O32	0.4708 (2)	0.1659 (3)	0.8178 (4)	0.0341 (11)	
O33	0.4791 (3)	0.1462 (4)	1.0216 (5)	0.0547 (16)	
O34	0.2843 (2)	0.1389 (3)	0.7637 (4)	0.0367 (11)	
O35	0.4338 (2)	0.5098 (3)	0.5822 (5)	0.0488 (15)	
O36	0.2790 (2)	0.3974 (3)	0.4560 (4)	0.0446 (14)	
N1	0.6761 (3)	0.4097 (4)	0.0486 (6)	0.0457 (17)	
N2	0.5963 (3)	0.3569 (4)	0.5746 (5)	0.0438 (16)	
N3	0.7647 (3)	0.3614 (4)	0.3484 (6)	0.0437 (16)	
N4	0.5971 (3)	0.1673 (3)	−0.0459 (5)	0.0390 (15)	
N5	0.4821 (3)	0.3901 (4)	0.2356 (6)	0.0476 (18)	
O47	0.6329 (3)	0.1665 (4)	0.4103 (5)	0.0617 (19)	
N7	0.6224 (3)	0.0801 (4)	0.5252 (5)	0.0400 (15)	
N8	0.4832 (3)	0.1029 (4)	0.2582 (6)	0.0474 (17)	
N9	0.7415 (3)	0.0838 (4)	0.1778 (6)	0.0471 (17)	
O37	0.2743 (2)	0.2842 (3)	0.8971 (4)	0.0382 (12)	
O38	0.2989 (3)	0.2710 (4)	1.1019 (4)	0.0632 (19)	
O39	0.3086 (3)	0.0201 (3)	0.8810 (5)	0.0559 (17)	
O55	0.5523 (2)	0.1732 (3)	0.6856 (5)	0.0459 (14)	
O40	0.6550 (2)	0.3481 (3)	0.1859 (5)	0.0490 (14)	
C2	0.6452 (3)	0.3714 (4)	0.1012 (7)	0.046 (2)	
H8	0.6142	0.3609	0.0743	0.055*	
C3	0.7237 (4)	0.4299 (5)	0.0907 (9)	0.061 (3)	
H2	0.7185	0.4556	0.1520	0.092*	
H3	0.7412	0.4606	0.0448	0.092*	
H4	0.7429	0.3867	0.1032	0.092*	
C4	0.6638 (5)	0.4329 (6)	−0.0517 (7)	0.067 (3)	
H6	0.6861	0.4101	−0.0980	0.101*	
H7	0.6670	0.4849	−0.0564	0.101*	
H5	0.6303	0.4191	−0.0668	0.101*	
O41	0.6001 (3)	0.3175 (4)	0.4158 (5)	0.0580 (17)	
C6	0.6147 (3)	0.3158 (5)	0.5037 (7)	0.051 (2)	
H10	0.6400	0.2836	0.5200	0.062*	
C7	0.6139 (4)	0.3522 (7)	0.6749 (7)	0.067 (3)	
H12	0.6340	0.3092	0.6823	0.100*	
H13	0.5861	0.3496	0.7195	0.100*	
H11	0.6335	0.3945	0.6902	0.100*	
C8	0.5581 (4)	0.4096 (6)	0.5489 (8)	0.068 (3)	
H15	0.5724	0.4481	0.5099	0.102*	
H16	0.5441	0.4297	0.6086	0.102*	
H14	0.5324	0.3860	0.5112	0.102*	
O42	0.7020 (2)	0.2798 (3)	0.3417 (5)	0.0472 (14)	
C10	0.7180 (3)	0.3417 (4)	0.3558 (6)	0.0430 (18)	
H18	0.6951	0.3774	0.3731	0.052*	
C11	0.8025 (4)	0.3079 (6)	0.3206 (8)	0.062 (3)	
H19	0.7881	0.2600	0.3203	0.093*	
H20	0.8293	0.3093	0.3678	0.093*	
H21	0.8151	0.3190	0.2555	0.093*	
C12	0.7807 (4)	0.4351 (6)	0.3660 (9)	0.067 (3)	
H22	0.7522	0.4657	0.3761	0.100*	
H23	0.7992	0.4523	0.3096	0.100*	
H24	0.8014	0.4365	0.4241	0.100*	
O43	0.6047 (3)	0.2262 (3)	0.0990 (4)	0.0514 (15)	
C14	0.5960 (3)	0.1715 (4)	0.0515 (6)	0.0422 (18)	
H26	0.5881	0.1294	0.0866	0.051*	
C15	0.5833 (4)	0.1013 (4)	−0.0986 (6)	0.046 (2)	
H28	0.5798	0.0620	−0.0519	0.069*	
H29	0.6087	0.0894	−0.1461	0.069*	
H27	0.5523	0.1088	−0.1325	0.069*	
C16	0.6085 (4)	0.2314 (5)	−0.1036 (7)	0.055 (2)	
H31	0.5807	0.2641	−0.1021	0.082*	
H30	0.6153	0.2175	−0.1710	0.082*	
H32	0.6372	0.2551	−0.0759	0.082*	
O44	0.5498 (2)	0.3180 (4)	0.2319 (5)	0.0528 (16)	
C18	0.5248 (3)	0.3674 (4)	0.2680 (8)	0.0468 (19)	
H34	0.5377	0.3907	0.3237	0.056*	
C19	0.4532 (4)	0.4425 (5)	0.2893 (9)	0.065 (3)	
H35	0.4220	0.4211	0.3081	0.098*	
H36	0.4472	0.4842	0.2479	0.098*	
H37	0.4709	0.4573	0.3478	0.098*	
C20	0.4613 (6)	0.3597 (9)	0.1481 (9)	0.114 (6)	
H39	0.4498	0.3982	0.1055	0.171*	
H40	0.4338	0.3289	0.1656	0.171*	
H38	0.4861	0.3316	0.1140	0.171*	
C21	0.6486 (4)	0.1287 (5)	0.4775 (6)	0.0453 (19)	
H47	0.6816	0.1349	0.4962	0.054*	
C22	0.5722 (3)	0.0645 (5)	0.4960 (9)	0.058 (2)	
H41	0.5499	0.0791	0.5481	0.087*	
H42	0.5687	0.0132	0.4841	0.087*	
H43	0.5645	0.0909	0.4364	0.087*	
C23	0.6432 (4)	0.0379 (6)	0.6040 (8)	0.064 (3)	
H44	0.6776	0.0503	0.6120	0.096*	
H46	0.6403	−0.0130	0.5884	0.096*	
H45	0.6257	0.0480	0.6646	0.096*	
O45	0.5562 (2)	0.1600 (3)	0.2673 (5)	0.0503 (14)	
C25	0.5106 (3)	0.1617 (4)	0.2671 (7)	0.0449 (19)	
H55	0.4948	0.2066	0.2735	0.054*	
C26	0.5044 (5)	0.0336 (5)	0.2361 (11)	0.088 (4)	
H49	0.5385	0.0398	0.2165	0.133*	
H51	0.4862	0.0111	0.1830	0.133*	
H50	0.5029	0.0031	0.2941	0.133*	
C27	0.4286 (4)	0.1112 (7)	0.2546 (9)	0.080 (4)	
H52	0.4201	0.1618	0.2609	0.120*	
H53	0.4139	0.0842	0.3081	0.120*	
H54	0.4164	0.0930	0.1923	0.120*	
C28	0.7155 (4)	0.1265 (5)	0.2333 (7)	0.050 (2)	
H56	0.7275	0.1363	0.2968	0.060*	
C29	0.7248 (4)	0.0661 (8)	0.0795 (9)	0.084 (4)	
H58	0.7093	0.1081	0.0501	0.126*	
H59	0.7012	0.0270	0.0829	0.126*	
H57	0.7526	0.0514	0.0396	0.126*	
C30	0.7896 (5)	0.0544 (8)	0.2084 (9)	0.088 (4)	
H61	0.8144	0.0676	0.1603	0.132*	
H62	0.7875	0.0023	0.2129	0.132*	
H60	0.7985	0.0741	0.2721	0.132*	
O52	0.3886 (3)	0.0253 (3)	0.5521 (5)	0.0571 (17)	

### Atomic displacement parameters (Å^2^)

**Table:**

	*U*^11^	*U*^22^	*U*^33^	*U*^12^	*U*^13^	*U*^23^
Eu1	0.03342 (16)	0.02974 (14)	0.03036 (15)	−0.00241 (13)	−0.00034 (15)	−0.00084 (15)
Mo1	0.0306 (3)	0.0302 (3)	0.0392 (3)	−0.0058 (2)	−0.0060 (3)	−0.0007 (3)
Mo2	0.0306 (3)	0.0352 (3)	0.0352 (3)	0.0008 (2)	−0.0079 (3)	0.0104 (3)
Mo3	0.0381 (3)	0.0250 (3)	0.0385 (3)	−0.0063 (2)	−0.0028 (3)	0.0059 (3)
Mo4	0.0258 (3)	0.0310 (3)	0.0333 (3)	0.0000 (2)	−0.0034 (2)	−0.0058 (3)
Mo5	0.0281 (3)	0.0369 (3)	0.0347 (3)	0.0098 (2)	−0.0026 (3)	−0.0085 (3)
Mo6	0.0397 (3)	0.0252 (3)	0.0403 (3)	0.0005 (2)	0.0023 (3)	−0.0095 (3)
Mo7	0.0455 (4)	0.0297 (3)	0.0399 (4)	−0.0055 (3)	0.0003 (3)	0.0121 (3)
Mo8	0.0409 (4)	0.0384 (3)	0.0306 (3)	0.0043 (3)	−0.0081 (3)	0.0091 (3)
Mo9	0.0560 (4)	0.0450 (4)	0.0268 (3)	0.0115 (3)	0.0128 (3)	0.0052 (3)
Mo10	0.0317 (3)	0.0291 (3)	0.0447 (4)	0.0035 (2)	0.0021 (3)	−0.0131 (3)
Mo11	0.0238 (3)	0.0406 (3)	0.0419 (4)	0.0031 (2)	0.0032 (3)	−0.0048 (3)
Mo12	0.0406 (3)	0.0386 (3)	0.0238 (3)	0.0044 (3)	0.0064 (3)	0.0030 (3)
P1	0.0222 (7)	0.0199 (6)	0.0198 (7)	0.0017 (5)	−0.0004 (5)	0.0009 (5)
O1	0.050 (4)	0.057 (4)	0.051 (4)	0.014 (3)	−0.006 (3)	−0.014 (3)
O2	0.040 (3)	0.040 (3)	0.056 (4)	−0.009 (2)	−0.007 (3)	−0.006 (3)
O3	0.034 (3)	0.036 (3)	0.035 (3)	0.000 (2)	−0.009 (2)	0.001 (2)
O4	0.034 (3)	0.027 (2)	0.035 (3)	0.0005 (19)	−0.003 (2)	0.005 (2)
O5	0.032 (3)	0.029 (2)	0.037 (3)	−0.0049 (19)	0.000 (2)	−0.002 (2)
O6	0.031 (3)	0.043 (3)	0.052 (3)	−0.004 (2)	−0.013 (2)	−0.009 (3)
O7	0.030 (3)	0.032 (2)	0.037 (3)	0.0019 (19)	0.002 (2)	−0.004 (2)
O8	0.038 (3)	0.027 (2)	0.042 (3)	0.008 (2)	0.002 (2)	−0.007 (2)
O9	0.035 (3)	0.031 (2)	0.033 (3)	0.001 (2)	−0.005 (2)	−0.006 (2)
O10	0.044 (3)	0.029 (2)	0.039 (3)	0.004 (2)	0.000 (2)	0.001 (2)
O11	0.051 (3)	0.032 (3)	0.040 (3)	0.004 (2)	−0.005 (3)	0.009 (2)
O12	0.037 (3)	0.035 (3)	0.032 (3)	0.004 (2)	0.001 (2)	0.000 (2)
O13	0.054 (3)	0.051 (3)	0.021 (2)	0.007 (3)	−0.003 (2)	0.004 (2)
O14	0.043 (3)	0.040 (3)	0.030 (3)	0.006 (2)	0.002 (2)	−0.003 (2)
O15	0.029 (3)	0.032 (2)	0.040 (3)	0.003 (2)	0.002 (2)	−0.003 (2)
O16	0.032 (3)	0.061 (4)	0.065 (4)	0.007 (3)	0.011 (3)	−0.009 (3)
O17	0.031 (3)	0.034 (2)	0.044 (3)	0.007 (2)	−0.002 (2)	0.005 (2)
O18	0.026 (3)	0.040 (3)	0.047 (3)	−0.004 (2)	0.003 (2)	0.000 (2)
O19	0.025 (2)	0.0205 (19)	0.024 (2)	0.0017 (16)	−0.0012 (17)	0.0037 (17)
O20	0.031 (2)	0.023 (2)	0.023 (2)	0.0016 (17)	0.0007 (18)	0.0042 (18)
O21	0.027 (2)	0.0232 (18)	0.022 (2)	0.0028 (16)	0.0001 (19)	−0.0043 (18)
O22	0.030 (2)	0.0222 (19)	0.022 (2)	0.0018 (17)	−0.0004 (18)	−0.0021 (17)
O23	0.035 (3)	0.038 (3)	0.031 (3)	0.002 (2)	−0.003 (2)	−0.001 (2)
O24	0.064 (4)	0.061 (4)	0.028 (3)	0.005 (3)	0.016 (3)	0.006 (3)
O25	0.032 (3)	0.035 (2)	0.036 (3)	−0.001 (2)	0.000 (2)	−0.003 (2)
O26	0.029 (2)	0.039 (3)	0.033 (3)	0.006 (2)	0.007 (2)	−0.004 (2)
O27	0.042 (3)	0.038 (3)	0.025 (2)	0.006 (2)	−0.002 (2)	−0.008 (2)
O28	0.050 (4)	0.038 (3)	0.062 (4)	0.006 (3)	0.004 (3)	−0.024 (3)
O29	0.038 (3)	0.0220 (19)	0.048 (3)	0.0043 (19)	−0.001 (2)	−0.002 (2)
O30	0.032 (3)	0.035 (2)	0.036 (3)	0.005 (2)	−0.003 (2)	−0.013 (2)
O31	0.051 (4)	0.039 (3)	0.042 (3)	−0.002 (2)	0.014 (3)	0.013 (2)
O32	0.042 (3)	0.030 (2)	0.030 (2)	0.011 (2)	−0.002 (2)	0.001 (2)
O33	0.060 (4)	0.061 (4)	0.043 (3)	0.013 (3)	−0.018 (3)	0.010 (3)
O34	0.041 (3)	0.029 (2)	0.039 (3)	−0.005 (2)	0.005 (2)	0.006 (2)
O35	0.054 (4)	0.038 (3)	0.055 (4)	−0.012 (3)	−0.002 (3)	0.014 (3)
O36	0.047 (3)	0.047 (3)	0.040 (3)	0.001 (3)	−0.016 (3)	0.015 (3)
N1	0.060 (5)	0.033 (3)	0.044 (4)	−0.002 (3)	0.008 (4)	0.000 (3)
N2	0.040 (4)	0.056 (4)	0.035 (3)	0.000 (3)	0.004 (3)	−0.007 (3)
N3	0.040 (4)	0.041 (3)	0.050 (4)	−0.002 (3)	−0.010 (3)	−0.007 (3)
N4	0.041 (4)	0.034 (3)	0.043 (4)	0.005 (3)	−0.008 (3)	−0.001 (3)
N5	0.049 (4)	0.039 (3)	0.054 (4)	0.007 (3)	0.000 (3)	0.012 (3)
O47	0.072 (5)	0.053 (4)	0.060 (4)	−0.010 (3)	−0.008 (4)	0.023 (3)
N7	0.043 (4)	0.037 (3)	0.040 (4)	−0.005 (3)	0.004 (3)	−0.002 (3)
N8	0.045 (4)	0.047 (4)	0.050 (4)	−0.016 (3)	0.006 (3)	−0.013 (3)
N9	0.052 (4)	0.043 (4)	0.047 (4)	0.005 (3)	0.002 (3)	0.001 (3)
O37	0.040 (3)	0.038 (3)	0.036 (3)	0.003 (2)	0.011 (2)	0.003 (2)
O38	0.075 (5)	0.085 (5)	0.030 (3)	0.017 (4)	0.026 (3)	0.003 (3)
O39	0.072 (4)	0.034 (3)	0.061 (4)	−0.017 (3)	−0.003 (3)	0.017 (3)
O55	0.036 (3)	0.054 (3)	0.048 (3)	0.017 (3)	−0.003 (3)	−0.015 (3)
O40	0.059 (4)	0.041 (3)	0.047 (3)	−0.016 (3)	−0.004 (3)	0.011 (3)
C2	0.049 (5)	0.029 (3)	0.060 (6)	−0.006 (3)	−0.002 (4)	−0.003 (4)
C3	0.051 (6)	0.053 (5)	0.079 (7)	−0.004 (4)	0.017 (5)	0.007 (5)
C4	0.105 (9)	0.059 (6)	0.037 (5)	−0.012 (6)	0.002 (5)	0.008 (5)
O41	0.057 (4)	0.077 (4)	0.040 (3)	0.019 (4)	−0.004 (3)	−0.016 (3)
C6	0.050 (5)	0.058 (5)	0.046 (5)	0.014 (4)	−0.006 (4)	−0.009 (4)
C7	0.062 (7)	0.103 (9)	0.036 (5)	−0.012 (6)	0.012 (5)	−0.015 (5)
C8	0.067 (7)	0.080 (7)	0.056 (6)	0.019 (6)	0.018 (5)	−0.007 (6)
O42	0.041 (3)	0.045 (3)	0.056 (4)	−0.006 (3)	−0.012 (3)	−0.001 (3)
C10	0.040 (4)	0.042 (4)	0.046 (5)	0.000 (3)	−0.003 (4)	−0.005 (4)
C11	0.047 (6)	0.074 (7)	0.066 (6)	0.006 (5)	−0.002 (5)	−0.018 (5)
C12	0.057 (6)	0.065 (6)	0.078 (7)	−0.023 (5)	−0.004 (6)	−0.009 (6)
O43	0.073 (4)	0.047 (3)	0.033 (3)	0.003 (3)	−0.010 (3)	−0.007 (3)
C14	0.053 (5)	0.039 (4)	0.034 (4)	0.003 (4)	−0.010 (4)	0.000 (4)
C15	0.061 (6)	0.040 (4)	0.038 (4)	0.011 (4)	−0.013 (4)	−0.003 (3)
C16	0.078 (7)	0.042 (4)	0.045 (5)	0.005 (5)	−0.003 (5)	0.004 (4)
O44	0.052 (4)	0.058 (4)	0.049 (4)	0.019 (3)	−0.002 (3)	0.000 (3)
C18	0.045 (5)	0.042 (4)	0.053 (5)	−0.003 (3)	−0.008 (4)	0.000 (4)
C19	0.058 (6)	0.044 (5)	0.093 (8)	0.007 (4)	0.007 (6)	0.016 (5)
C20	0.128 (13)	0.149 (14)	0.064 (8)	0.055 (11)	−0.058 (9)	−0.021 (9)
C21	0.049 (5)	0.045 (4)	0.042 (5)	−0.010 (4)	0.001 (4)	0.007 (4)
C22	0.050 (5)	0.044 (5)	0.081 (7)	−0.011 (4)	0.005 (5)	−0.006 (5)
C23	0.075 (7)	0.065 (6)	0.051 (6)	−0.018 (5)	−0.011 (5)	0.023 (5)
O45	0.041 (3)	0.046 (3)	0.064 (4)	−0.013 (3)	−0.001 (3)	0.008 (3)
C25	0.050 (5)	0.039 (4)	0.046 (5)	−0.014 (3)	0.003 (4)	−0.007 (4)
C26	0.084 (9)	0.041 (5)	0.140 (13)	−0.009 (6)	0.007 (8)	−0.008 (6)
C27	0.052 (6)	0.106 (9)	0.083 (9)	−0.023 (6)	0.014 (6)	−0.027 (7)
C28	0.065 (6)	0.044 (4)	0.042 (5)	0.009 (4)	0.001 (4)	−0.013 (4)
C29	0.070 (8)	0.119 (10)	0.063 (7)	0.003 (7)	0.001 (6)	−0.047 (7)
C30	0.095 (10)	0.100 (9)	0.069 (8)	0.057 (8)	−0.008 (7)	−0.006 (7)
O52	0.069 (4)	0.035 (3)	0.067 (4)	−0.004 (3)	0.007 (4)	−0.025 (3)

### Geometric parameters (Å, º)

**Table:**

Eu1—O40	2.369 (5)	N1—C3	1.450 (12)
Eu1—O47	2.378 (6)	N1—C4	1.452 (12)
Eu1—O1	2.383 (6)	N2—C6	1.311 (11)
Eu1—O45	2.387 (5)	N2—C7	1.433 (12)
Eu1—O41	2.395 (6)	N2—C8	1.455 (12)
Eu1—O44	2.413 (6)	N3—C10	1.312 (11)
Eu1—O42	2.415 (6)	N3—C12	1.438 (11)
Eu1—O43	2.416 (6)	N3—C11	1.463 (12)
Mo1—O2	1.672 (5)	N4—C14	1.313 (10)
Mo1—O9	1.836 (5)	N4—C16	1.441 (11)
Mo1—O3	1.852 (5)	N4—C15	1.452 (10)
Mo1—O34	1.973 (5)	N5—C18	1.297 (11)
Mo1—O18	1.986 (5)	N5—C20	1.417 (14)
Mo1—O19	2.436 (4)	N5—C19	1.433 (12)
Mo2—O36	1.690 (5)	O47—C21	1.214 (11)
Mo2—O4	1.831 (5)	N7—C21	1.307 (10)
Mo2—O17	1.866 (5)	N7—C23	1.427 (12)
Mo2—O23	1.957 (5)	N7—C22	1.435 (11)
Mo2—O3	1.993 (5)	N8—C25	1.313 (10)
Mo2—O19	2.429 (4)	N8—C26	1.427 (13)
Mo3—O35	1.672 (5)	N8—C27	1.477 (13)
Mo3—O25	1.842 (5)	N9—C28	1.290 (11)
Mo3—O5	1.848 (5)	N9—C29	1.435 (13)
Mo3—O4	1.985 (5)	N9—C30	1.461 (14)
Mo3—O29	1.985 (5)	O40—C2	1.245 (11)
Mo3—O21	2.428 (4)	C2—H8	0.9300
Mo4—O6	1.677 (5)	C3—H2	0.9600
Mo4—O12	1.834 (5)	C3—H3	0.9600
Mo4—O30	1.851 (5)	C3—H4	0.9600
Mo4—O5	1.976 (5)	C4—H6	0.9600
Mo4—O7	1.992 (5)	C4—H7	0.9600
Mo4—O21	2.426 (4)	C4—H5	0.9600
Mo5—O55	1.675 (5)	O41—C6	1.245 (11)
Mo5—O7	1.825 (5)	C6—H10	0.9300
Mo5—O26	1.845 (5)	C7—H12	0.9600
Mo5—O32	1.979 (5)	C7—H13	0.9600
Mo5—O8	1.985 (5)	C7—H11	0.9600
Mo5—O22	2.428 (5)	C8—H15	0.9600
Mo6—O52	1.676 (5)	C8—H16	0.9600
Mo6—O10	1.837 (5)	C8—H14	0.9600
Mo6—O8	1.852 (5)	O42—C10	1.228 (9)
Mo6—O9	1.983 (5)	C10—H18	0.9300
Mo6—O27	2.000 (5)	C11—H19	0.9600
Mo6—O22	2.416 (4)	C11—H20	0.9600
Mo7—O39	1.686 (5)	C11—H21	0.9600
Mo7—O34	1.842 (6)	C12—H22	0.9600
Mo7—O31	1.854 (6)	C12—H23	0.9600
Mo7—O10	1.973 (6)	C12—H24	0.9600
Mo7—O11	1.994 (6)	O43—C14	1.214 (10)
Mo7—O20	2.438 (4)	C14—H26	0.9300
Mo8—O33	1.675 (6)	C15—H28	0.9600
Mo8—O11	1.844 (6)	C15—H29	0.9600
Mo8—O32	1.853 (5)	C15—H27	0.9600
Mo8—O12	1.983 (5)	C16—H31	0.9600
Mo8—O13	1.987 (6)	C16—H30	0.9600
Mo8—O20	2.423 (4)	C16—H32	0.9600
Mo9—O38	1.680 (6)	O44—C18	1.229 (10)
Mo9—O14	1.838 (6)	C18—H34	0.9300
Mo9—O13	1.851 (6)	C19—H35	0.9600
Mo9—O31	1.984 (6)	C19—H36	0.9600
Mo9—O37	1.993 (6)	C19—H37	0.9600
Mo9—O20	2.433 (5)	C20—H39	0.9600
Mo10—O28	1.690 (5)	C20—H40	0.9600
Mo10—O15	1.842 (5)	C20—H38	0.9600
Mo10—O29	1.847 (6)	C21—H47	0.9300
Mo10—O14	1.976 (5)	C22—H41	0.9600
Mo10—O30	1.984 (5)	C22—H42	0.9600
Mo10—O21	2.420 (4)	C22—H43	0.9600
Mo11—O16	1.676 (6)	C23—H44	0.9600
Mo11—O37	1.836 (5)	C23—H46	0.9600
Mo11—O18	1.857 (5)	C23—H45	0.9600
Mo11—O15	1.976 (5)	O45—C25	1.227 (10)
Mo11—O17	1.979 (5)	C25—H55	0.9300
Mo11—O19	2.423 (4)	C26—H49	0.9600
Mo12—O24	1.684 (5)	C26—H51	0.9600
Mo12—O23	1.840 (5)	C26—H50	0.9600
Mo12—O27	1.850 (5)	C27—H52	0.9600
Mo12—O25	1.976 (5)	C27—H53	0.9600
Mo12—O26	1.980 (5)	C27—H54	0.9600
Mo12—O22	2.428 (4)	C28—H56	0.9300
P1—O19	1.521 (5)	C29—H58	0.9600
P1—O20	1.526 (5)	C29—H59	0.9600
P1—O22	1.527 (5)	C29—H57	0.9600
P1—O21	1.536 (4)	C30—H61	0.9600
O1—C28	1.219 (11)	C30—H62	0.9600
N1—C2	1.297 (11)	C30—H60	0.9600
			
O40—Eu1—O47	145.6 (2)	O26—Mo12—O22	72.45 (18)
O40—Eu1—O1	98.6 (2)	O19—P1—O20	109.8 (3)
O47—Eu1—O1	76.7 (2)	O19—P1—O22	109.5 (3)
O40—Eu1—O45	141.7 (2)	O20—P1—O22	109.1 (2)
O47—Eu1—O45	72.7 (2)	O19—P1—O21	109.3 (2)
O1—Eu1—O45	88.5 (2)	O20—P1—O21	109.5 (3)
O40—Eu1—O41	93.6 (2)	O22—P1—O21	109.6 (3)
O47—Eu1—O41	75.1 (3)	C28—O1—Eu1	137.4 (6)
O1—Eu1—O41	145.4 (2)	Mo1—O3—Mo2	124.5 (3)
O45—Eu1—O41	101.7 (2)	Mo2—O4—Mo3	151.4 (3)
O40—Eu1—O44	76.2 (2)	Mo3—O5—Mo4	125.8 (3)
O47—Eu1—O44	127.6 (2)	Mo5—O7—Mo4	151.7 (3)
O1—Eu1—O44	142.6 (2)	Mo6—O8—Mo5	124.8 (2)
O45—Eu1—O44	75.6 (2)	Mo1—O9—Mo6	151.6 (3)
O41—Eu1—O44	71.8 (2)	Mo6—O10—Mo7	151.5 (3)
O40—Eu1—O42	70.2 (2)	Mo8—O11—Mo7	125.5 (3)
O47—Eu1—O42	75.6 (2)	Mo4—O12—Mo8	152.1 (3)
O1—Eu1—O42	76.8 (2)	Mo9—O13—Mo8	124.6 (3)
O45—Eu1—O42	147.4 (2)	Mo9—O14—Mo10	151.8 (3)
O41—Eu1—O42	77.2 (2)	Mo10—O15—Mo11	151.4 (3)
O44—Eu1—O42	132.0 (2)	Mo2—O17—Mo11	124.8 (3)
O40—Eu1—O43	73.5 (2)	Mo11—O18—Mo1	124.6 (3)
O47—Eu1—O43	132.8 (2)	P1—O19—Mo11	126.2 (2)
O1—Eu1—O43	70.2 (2)	P1—O19—Mo2	125.7 (2)
O45—Eu1—O43	73.7 (2)	Mo11—O19—Mo2	89.23 (14)
O41—Eu1—O43	144.3 (2)	P1—O19—Mo1	126.1 (2)
O44—Eu1—O43	72.8 (2)	Mo11—O19—Mo1	88.93 (15)
O42—Eu1—O43	125.7 (2)	Mo2—O19—Mo1	88.80 (15)
O2—Mo1—O9	102.7 (3)	P1—O20—Mo8	126.5 (3)
O2—Mo1—O3	102.4 (3)	P1—O20—Mo9	125.6 (2)
O9—Mo1—O3	95.8 (2)	Mo8—O20—Mo9	88.87 (15)
O2—Mo1—O34	101.0 (3)	P1—O20—Mo7	125.7 (3)
O9—Mo1—O34	85.0 (2)	Mo8—O20—Mo7	89.20 (14)
O3—Mo1—O34	155.8 (2)	Mo9—O20—Mo7	89.09 (15)
O2—Mo1—O18	100.5 (3)	P1—O21—Mo10	126.1 (3)
O9—Mo1—O18	155.7 (2)	P1—O21—Mo4	125.6 (2)
O3—Mo1—O18	86.5 (2)	Mo10—O21—Mo4	89.12 (14)
O34—Mo1—O18	83.2 (2)	P1—O21—Mo3	126.0 (3)
O2—Mo1—O19	171.9 (2)	Mo10—O21—Mo3	89.13 (13)
O9—Mo1—O19	85.20 (18)	Mo4—O21—Mo3	89.11 (14)
O3—Mo1—O19	74.36 (18)	P1—O22—Mo6	126.0 (3)
O34—Mo1—O19	81.65 (18)	P1—O22—Mo5	126.1 (3)
O18—Mo1—O19	72.08 (18)	Mo6—O22—Mo5	89.19 (14)
O36—Mo2—O4	103.7 (2)	P1—O22—Mo12	126.0 (2)
O36—Mo2—O17	102.2 (3)	Mo6—O22—Mo12	89.10 (14)
O4—Mo2—O17	95.3 (2)	Mo5—O22—Mo12	88.42 (15)
O36—Mo2—O23	101.5 (3)	Mo12—O23—Mo2	152.1 (3)
O4—Mo2—O23	85.7 (2)	Mo3—O25—Mo12	152.5 (3)
O17—Mo2—O23	155.4 (2)	Mo5—O26—Mo12	124.5 (3)
O36—Mo2—O3	98.6 (3)	Mo12—O27—Mo6	123.9 (3)
O4—Mo2—O3	156.8 (2)	Mo10—O29—Mo3	125.1 (2)
O17—Mo2—O3	86.4 (2)	Mo4—O30—Mo10	124.8 (3)
O23—Mo2—O3	83.5 (2)	Mo7—O31—Mo9	125.8 (3)
O36—Mo2—O19	170.1 (2)	Mo8—O32—Mo5	151.7 (3)
O4—Mo2—O19	85.87 (18)	Mo7—O34—Mo1	152.0 (3)
O17—Mo2—O19	73.79 (19)	C2—N1—C3	119.4 (8)
O23—Mo2—O19	81.74 (18)	C2—N1—C4	121.4 (9)
O3—Mo2—O19	72.30 (18)	C3—N1—C4	119.2 (8)
O35—Mo3—O25	102.3 (3)	C6—N2—C7	121.7 (8)
O35—Mo3—O5	102.5 (3)	C6—N2—C8	118.4 (8)
O25—Mo3—O5	96.1 (2)	C7—N2—C8	119.9 (8)
O35—Mo3—O4	102.0 (3)	C10—N3—C12	122.2 (8)
O25—Mo3—O4	84.4 (2)	C10—N3—C11	120.0 (7)
O5—Mo3—O4	154.8 (2)	C12—N3—C11	117.8 (8)
O35—Mo3—O29	100.9 (3)	C14—N4—C16	119.6 (7)
O25—Mo3—O29	155.3 (2)	C14—N4—C15	122.0 (7)
O5—Mo3—O29	87.0 (2)	C16—N4—C15	118.2 (7)
O4—Mo3—O29	82.7 (2)	C18—N5—C20	120.2 (9)
O35—Mo3—O21	171.5 (2)	C18—N5—C19	121.8 (9)
O25—Mo3—O21	85.71 (19)	C20—N5—C19	117.9 (10)
O5—Mo3—O21	73.44 (19)	C21—O47—Eu1	166.5 (7)
O4—Mo3—O21	81.45 (17)	C21—N7—C23	121.6 (8)
O29—Mo3—O21	71.66 (17)	C21—N7—C22	120.6 (8)
O6—Mo4—O12	103.5 (3)	C23—N7—C22	117.7 (8)
O6—Mo4—O30	103.7 (2)	C25—N8—C26	121.7 (9)
O12—Mo4—O30	96.0 (2)	C25—N8—C27	118.5 (9)
O6—Mo4—O5	99.6 (2)	C26—N8—C27	118.9 (9)
O12—Mo4—O5	155.3 (2)	C28—N9—C29	120.0 (9)
O30—Mo4—O5	87.0 (2)	C28—N9—C30	122.9 (9)
O6—Mo4—O7	100.0 (2)	C29—N9—C30	117.0 (9)
O12—Mo4—O7	85.3 (2)	Mo11—O37—Mo9	151.5 (3)
O30—Mo4—O7	155.2 (2)	C2—O40—Eu1	130.2 (5)
O5—Mo4—O7	82.0 (2)	O40—C2—N1	123.4 (9)
O6—Mo4—O21	170.8 (2)	O40—C2—H8	118.3
O12—Mo4—O21	85.6 (2)	N1—C2—H8	118.3
O30—Mo4—O21	73.97 (19)	N1—C3—H2	109.5
O5—Mo4—O21	71.54 (18)	N1—C3—H3	109.5
O7—Mo4—O21	81.47 (18)	H2—C3—H3	109.5
O55—Mo5—O7	103.4 (3)	N1—C3—H4	109.5
O55—Mo5—O26	102.0 (3)	H2—C3—H4	109.5
O7—Mo5—O26	96.1 (2)	H3—C3—H4	109.5
O55—Mo5—O32	101.0 (3)	N1—C4—H6	109.5
O7—Mo5—O32	85.1 (2)	N1—C4—H7	109.5
O26—Mo5—O32	156.0 (2)	H6—C4—H7	109.5
O55—Mo5—O8	99.6 (3)	N1—C4—H5	109.5
O7—Mo5—O8	155.5 (2)	H6—C4—H5	109.5
O26—Mo5—O8	87.2 (2)	H7—C4—H5	109.5
O32—Mo5—O8	82.3 (2)	C6—O41—Eu1	132.0 (6)
O55—Mo5—O22	170.7 (2)	O41—C6—N2	123.9 (9)
O7—Mo5—O22	85.67 (19)	O41—C6—H10	118.1
O26—Mo5—O22	74.59 (18)	N2—C6—H10	118.1
O32—Mo5—O22	81.61 (18)	N2—C7—H12	109.5
O8—Mo5—O22	71.80 (17)	N2—C7—H13	109.5
O52—Mo6—O10	103.3 (3)	H12—C7—H13	109.5
O52—Mo6—O8	102.5 (3)	N2—C7—H11	109.5
O10—Mo6—O8	95.8 (2)	H12—C7—H11	109.5
O52—Mo6—O9	100.9 (3)	H13—C7—H11	109.5
O10—Mo6—O9	85.6 (2)	N2—C8—H15	109.5
O8—Mo6—O9	155.5 (2)	N2—C8—H16	109.5
O52—Mo6—O27	99.1 (3)	H15—C8—H16	109.5
O10—Mo6—O27	156.2 (2)	N2—C8—H14	109.5
O8—Mo6—O27	87.2 (2)	H15—C8—H14	109.5
O9—Mo6—O27	82.1 (2)	H16—C8—H14	109.5
O52—Mo6—O22	170.8 (3)	C10—O42—Eu1	127.1 (5)
O10—Mo6—O22	85.69 (19)	O42—C10—N3	125.4 (8)
O8—Mo6—O22	74.14 (18)	O42—C10—H18	117.3
O9—Mo6—O22	81.56 (18)	N3—C10—H18	117.3
O27—Mo6—O22	72.40 (17)	N3—C11—H19	109.5
O39—Mo7—O34	103.4 (3)	N3—C11—H20	109.5
O39—Mo7—O31	102.8 (3)	H19—C11—H20	109.5
O34—Mo7—O31	96.1 (3)	N3—C11—H21	109.5
O39—Mo7—O10	102.0 (3)	H19—C11—H21	109.5
O34—Mo7—O10	85.3 (2)	H20—C11—H21	109.5
O31—Mo7—O10	154.1 (2)	N3—C12—H22	109.5
O39—Mo7—O11	99.8 (3)	N3—C12—H23	109.5
O34—Mo7—O11	155.4 (2)	H22—C12—H23	109.5
O31—Mo7—O11	86.4 (3)	N3—C12—H24	109.5
O10—Mo7—O11	82.1 (2)	H22—C12—H24	109.5
O39—Mo7—O20	170.4 (3)	H23—C12—H24	109.5
O34—Mo7—O20	85.93 (18)	C14—O43—Eu1	132.6 (5)
O31—Mo7—O20	73.5 (2)	O43—C14—N4	124.7 (8)
O10—Mo7—O20	80.88 (19)	O43—C14—H26	117.6
O11—Mo7—O20	71.30 (18)	N4—C14—H26	117.6
O33—Mo8—O11	102.1 (3)	N4—C15—H28	109.5
O33—Mo8—O32	103.5 (3)	N4—C15—H29	109.5
O11—Mo8—O32	96.5 (2)	H28—C15—H29	109.5
O33—Mo8—O12	101.9 (3)	N4—C15—H27	109.5
O11—Mo8—O12	155.0 (2)	H28—C15—H27	109.5
O32—Mo8—O12	84.6 (2)	H29—C15—H27	109.5
O33—Mo8—O13	98.9 (3)	N4—C16—H31	109.5
O11—Mo8—O13	86.8 (3)	N4—C16—H30	109.5
O32—Mo8—O13	156.0 (2)	H31—C16—H30	109.5
O12—Mo8—O13	82.7 (2)	N4—C16—H32	109.5
O33—Mo8—O20	170.4 (3)	H31—C16—H32	109.5
O11—Mo8—O20	74.0 (2)	H30—C16—H32	109.5
O32—Mo8—O20	85.74 (19)	C18—O44—Eu1	138.8 (6)
O12—Mo8—O20	81.23 (18)	O44—C18—N5	126.1 (9)
O13—Mo8—O20	72.31 (19)	O44—C18—H34	117.0
O38—Mo9—O14	103.1 (3)	N5—C18—H34	117.0
O38—Mo9—O13	102.6 (3)	N5—C19—H35	109.5
O14—Mo9—O13	96.7 (3)	N5—C19—H36	109.5
O38—Mo9—O31	99.6 (3)	H35—C19—H36	109.5
O14—Mo9—O31	155.6 (2)	N5—C19—H37	109.5
O13—Mo9—O31	86.9 (3)	H35—C19—H37	109.5
O38—Mo9—O37	101.1 (3)	H36—C19—H37	109.5
O14—Mo9—O37	85.0 (2)	N5—C20—H39	109.5
O13—Mo9—O37	155.1 (2)	N5—C20—H40	109.5
O31—Mo9—O37	81.9 (2)	H39—C20—H40	109.5
O38—Mo9—O20	170.6 (3)	N5—C20—H38	109.5
O14—Mo9—O20	86.07 (19)	H39—C20—H38	109.5
O13—Mo9—O20	74.19 (19)	H40—C20—H38	109.5
O31—Mo9—O20	71.60 (19)	O47—C21—N7	124.6 (9)
O37—Mo9—O20	81.18 (18)	O47—C21—H47	117.7
O28—Mo10—O15	103.4 (3)	N7—C21—H47	117.7
O28—Mo10—O29	103.0 (3)	N7—C22—H41	109.5
O15—Mo10—O29	95.3 (2)	N7—C22—H42	109.5
O28—Mo10—O14	100.5 (3)	H41—C22—H42	109.5
O15—Mo10—O14	85.4 (2)	N7—C22—H43	109.5
O29—Mo10—O14	155.7 (2)	H41—C22—H43	109.5
O28—Mo10—O30	99.6 (3)	H42—C22—H43	109.5
O15—Mo10—O30	155.7 (2)	N7—C23—H44	109.5
O29—Mo10—O30	87.1 (2)	N7—C23—H46	109.5
O14—Mo10—O30	82.7 (2)	H44—C23—H46	109.5
O28—Mo10—O21	171.0 (2)	N7—C23—H45	109.5
O15—Mo10—O21	85.38 (19)	H44—C23—H45	109.5
O29—Mo10—O21	74.00 (18)	H46—C23—H45	109.5
O14—Mo10—O21	81.87 (18)	C25—O45—Eu1	136.7 (6)
O30—Mo10—O21	72.04 (17)	O45—C25—N8	122.8 (8)
O16—Mo11—O37	104.0 (3)	O45—C25—H55	118.6
O16—Mo11—O18	103.5 (3)	N8—C25—H55	118.6
O37—Mo11—O18	95.7 (2)	N8—C26—H49	109.5
O16—Mo11—O15	99.8 (3)	N8—C26—H51	109.5
O37—Mo11—O15	85.6 (2)	H49—C26—H51	109.5
O18—Mo11—O15	155.6 (2)	N8—C26—H50	109.5
O16—Mo11—O17	98.8 (3)	H49—C26—H50	109.5
O37—Mo11—O17	155.7 (2)	H51—C26—H50	109.5
O18—Mo11—O17	86.8 (2)	N8—C27—H52	109.5
O15—Mo11—O17	82.4 (2)	N8—C27—H53	109.5
O16—Mo11—O19	170.7 (3)	H52—C27—H53	109.5
O37—Mo11—O19	85.24 (19)	N8—C27—H54	109.5
O18—Mo11—O19	74.38 (19)	H52—C27—H54	109.5
O15—Mo11—O19	81.50 (18)	H53—C27—H54	109.5
O17—Mo11—O19	72.16 (18)	O1—C28—N9	125.3 (9)
O24—Mo12—O23	102.9 (3)	O1—C28—H56	117.4
O24—Mo12—O27	101.4 (3)	N9—C28—H56	117.4
O23—Mo12—O27	95.4 (2)	N9—C29—H58	109.5
O24—Mo12—O25	101.9 (3)	N9—C29—H59	109.5
O23—Mo12—O25	84.4 (2)	H58—C29—H59	109.5
O27—Mo12—O25	156.2 (2)	N9—C29—H57	109.5
O24—Mo12—O26	100.0 (3)	H58—C29—H57	109.5
O23—Mo12—O26	155.8 (2)	H59—C29—H57	109.5
O27—Mo12—O26	87.7 (2)	N9—C30—H61	109.5
O25—Mo12—O26	83.2 (2)	N9—C30—H62	109.5
O24—Mo12—O22	171.3 (3)	H61—C30—H62	109.5
O23—Mo12—O22	85.27 (19)	N9—C30—H60	109.5
O27—Mo12—O22	74.48 (18)	H61—C30—H60	109.5
O25—Mo12—O22	81.80 (18)	H62—C30—H60	109.5
			
O2—Mo1—O3—Mo2	171.0 (3)	O22—P1—O21—Mo10	175.5 (3)
O9—Mo1—O3—Mo2	−84.6 (3)	O19—P1—O21—Mo4	175.3 (3)
O34—Mo1—O3—Mo2	6.0 (8)	O20—P1—O21—Mo4	55.0 (4)
O18—Mo1—O3—Mo2	71.1 (3)	O22—P1—O21—Mo4	−64.6 (4)
O19—Mo1—O3—Mo2	−1.3 (3)	O19—P1—O21—Mo3	−64.9 (3)
O36—Mo2—O4—Mo3	−129.9 (6)	O20—P1—O21—Mo3	174.7 (3)
O17—Mo2—O4—Mo3	126.1 (6)	O22—P1—O21—Mo3	55.1 (3)
O23—Mo2—O4—Mo3	−29.1 (6)	O19—P1—O22—Mo6	−64.7 (3)
O3—Mo2—O4—Mo3	33.1 (10)	O20—P1—O22—Mo6	55.5 (4)
O19—Mo2—O4—Mo3	52.9 (6)	O21—P1—O22—Mo6	175.3 (3)
O35—Mo3—O5—Mo4	169.8 (3)	O19—P1—O22—Mo5	174.7 (3)
O25—Mo3—O5—Mo4	−86.2 (3)	O20—P1—O22—Mo5	−65.1 (3)
O4—Mo3—O5—Mo4	3.4 (7)	O21—P1—O22—Mo5	54.8 (4)
O29—Mo3—O5—Mo4	69.3 (3)	O19—P1—O22—Mo12	55.5 (4)
O21—Mo3—O5—Mo4	−2.5 (3)	O20—P1—O22—Mo12	175.7 (3)
O55—Mo5—O7—Mo4	−129.4 (6)	O21—P1—O22—Mo12	−64.4 (4)
O26—Mo5—O7—Mo4	126.7 (6)	O24—Mo12—O23—Mo2	−130.2 (6)
O32—Mo5—O7—Mo4	−29.2 (6)	O27—Mo12—O23—Mo2	126.8 (6)
O8—Mo5—O7—Mo4	30.1 (10)	O25—Mo12—O23—Mo2	−29.3 (6)
O22—Mo5—O7—Mo4	52.8 (6)	O26—Mo12—O23—Mo2	30.3 (10)
O52—Mo6—O8—Mo5	168.5 (4)	O22—Mo12—O23—Mo2	52.9 (6)
O10—Mo6—O8—Mo5	−86.5 (3)	O35—Mo3—O25—Mo12	−132.4 (7)
O9—Mo6—O8—Mo5	5.7 (8)	O5—Mo3—O25—Mo12	123.3 (7)
O27—Mo6—O8—Mo5	69.8 (3)	O4—Mo3—O25—Mo12	−31.3 (6)
O22—Mo6—O8—Mo5	−2.7 (3)	O29—Mo3—O25—Mo12	27.3 (10)
O2—Mo1—O9—Mo6	−129.2 (6)	O21—Mo3—O25—Mo12	50.5 (6)
O3—Mo1—O9—Mo6	126.6 (6)	O55—Mo5—O26—Mo12	169.1 (3)
O34—Mo1—O9—Mo6	−29.1 (6)	O7—Mo5—O26—Mo12	−85.8 (3)
O18—Mo1—O9—Mo6	32.2 (10)	O32—Mo5—O26—Mo12	5.8 (7)
O19—Mo1—O9—Mo6	52.9 (6)	O8—Mo5—O26—Mo12	69.8 (3)
O52—Mo6—O10—Mo7	−128.1 (7)	O22—Mo5—O26—Mo12	−2.0 (3)
O8—Mo6—O10—Mo7	127.5 (7)	O24—Mo12—O27—Mo6	169.1 (3)
O9—Mo6—O10—Mo7	−27.9 (7)	O23—Mo12—O27—Mo6	−86.6 (3)
O27—Mo6—O10—Mo7	31.2 (11)	O25—Mo12—O27—Mo6	1.8 (8)
O22—Mo6—O10—Mo7	54.0 (7)	O26—Mo12—O27—Mo6	69.4 (3)
O33—Mo8—O11—Mo7	168.8 (4)	O22—Mo12—O27—Mo6	−3.0 (3)
O32—Mo8—O11—Mo7	−85.8 (4)	O28—Mo10—O29—Mo3	168.8 (3)
O12—Mo8—O11—Mo7	5.2 (8)	O15—Mo10—O29—Mo3	−86.1 (3)
O13—Mo8—O11—Mo7	70.3 (4)	O14—Mo10—O29—Mo3	4.4 (8)
O20—Mo8—O11—Mo7	−2.2 (3)	O30—Mo10—O29—Mo3	69.6 (3)
O6—Mo4—O12—Mo8	−128.2 (6)	O21—Mo10—O29—Mo3	−2.5 (3)
O30—Mo4—O12—Mo8	126.1 (6)	O6—Mo4—O30—Mo10	168.6 (3)
O5—Mo4—O12—Mo8	30.3 (10)	O12—Mo4—O30—Mo10	−85.9 (4)
O7—Mo4—O12—Mo8	−29.0 (6)	O5—Mo4—O30—Mo10	69.4 (3)
O21—Mo4—O12—Mo8	52.8 (6)	O7—Mo4—O30—Mo10	5.8 (8)
O38—Mo9—O13—Mo8	169.0 (4)	O21—Mo4—O30—Mo10	−2.2 (3)
O14—Mo9—O13—Mo8	−85.8 (4)	O39—Mo7—O31—Mo9	168.4 (4)
O31—Mo9—O13—Mo8	69.9 (4)	O34—Mo7—O31—Mo9	−86.3 (4)
O37—Mo9—O13—Mo8	6.8 (9)	O10—Mo7—O31—Mo9	5.5 (8)
O20—Mo9—O13—Mo8	−1.9 (3)	O11—Mo7—O31—Mo9	69.2 (4)
O38—Mo9—O14—Mo10	−131.0 (7)	O20—Mo7—O31—Mo9	−2.4 (3)
O13—Mo9—O14—Mo10	124.3 (7)	O33—Mo8—O32—Mo5	−132.1 (7)
O31—Mo9—O14—Mo10	27.0 (11)	O11—Mo8—O32—Mo5	123.8 (7)
O37—Mo9—O14—Mo10	−30.7 (6)	O12—Mo8—O32—Mo5	−31.1 (7)
O20—Mo9—O14—Mo10	50.8 (6)	O13—Mo8—O32—Mo5	27.1 (11)
O28—Mo10—O15—Mo11	−128.4 (6)	O20—Mo8—O32—Mo5	50.5 (6)
O29—Mo10—O15—Mo11	126.9 (6)	O39—Mo7—O34—Mo1	−131.2 (6)
O14—Mo10—O15—Mo11	−28.7 (6)	O31—Mo7—O34—Mo1	124.0 (6)
O30—Mo10—O15—Mo11	32.1 (10)	O10—Mo7—O34—Mo1	−30.0 (6)
O21—Mo10—O15—Mo11	53.5 (6)	O11—Mo7—O34—Mo1	29.3 (10)
O36—Mo2—O17—Mo11	168.2 (3)	O20—Mo7—O34—Mo1	51.1 (6)
O4—Mo2—O17—Mo11	−86.5 (3)	O16—Mo11—O37—Mo9	−127.0 (6)
O23—Mo2—O17—Mo11	4.5 (7)	O18—Mo11—O37—Mo9	127.6 (6)
O3—Mo2—O17—Mo11	70.2 (3)	O15—Mo11—O37—Mo9	−28.0 (6)
O19—Mo2—O17—Mo11	−2.4 (3)	O17—Mo11—O37—Mo9	32.5 (10)
O16—Mo11—O18—Mo1	168.6 (4)	O19—Mo11—O37—Mo9	53.8 (6)
O37—Mo11—O18—Mo1	−85.5 (4)	Eu1—O40—C2—N1	−154.9 (6)
O15—Mo11—O18—Mo1	6.6 (8)	C3—N1—C2—O40	−3.1 (13)
O17—Mo11—O18—Mo1	70.3 (3)	C4—N1—C2—O40	177.2 (8)
O19—Mo11—O18—Mo1	−2.1 (3)	Eu1—O41—C6—N2	−176.3 (7)
O20—P1—O19—Mo11	55.7 (3)	C7—N2—C6—O41	179.1 (10)
O22—P1—O19—Mo11	175.5 (3)	C8—N2—C6—O41	−2.5 (15)
O21—P1—O19—Mo11	−64.4 (3)	Eu1—O42—C10—N3	−146.6 (7)
O20—P1—O19—Mo2	175.9 (3)	C12—N3—C10—O42	180.0 (9)
O22—P1—O19—Mo2	−64.3 (3)	C11—N3—C10—O42	0.5 (14)
O21—P1—O19—Mo2	55.8 (4)	Eu1—O43—C14—N4	−165.4 (6)
O20—P1—O19—Mo1	−64.6 (3)	C16—N4—C14—O43	−0.9 (14)
O22—P1—O19—Mo1	55.2 (3)	C15—N4—C14—O43	−176.1 (8)
O21—P1—O19—Mo1	175.3 (3)	Eu1—O44—C18—N5	−169.7 (7)
O19—P1—O20—Mo8	175.4 (3)	C20—N5—C18—O44	−3.2 (16)
O22—P1—O20—Mo8	55.4 (4)	C19—N5—C18—O44	172.7 (9)
O21—P1—O20—Mo8	−64.6 (4)	Eu1—O47—C21—N7	−167 (2)
O19—P1—O20—Mo9	−64.7 (4)	C23—N7—C21—O47	−179.5 (9)
O22—P1—O20—Mo9	175.3 (3)	C22—N7—C21—O47	4.0 (14)
O21—P1—O20—Mo9	55.3 (4)	Eu1—O45—C25—N8	176.5 (6)
O19—P1—O20—Mo7	54.7 (4)	C26—N8—C25—O45	−7.7 (16)
O22—P1—O20—Mo7	−65.4 (4)	C27—N8—C25—O45	−177.4 (9)
O21—P1—O20—Mo7	174.7 (3)	Eu1—O1—C28—N9	−169.7 (7)
O19—P1—O21—Mo10	55.4 (4)	C29—N9—C28—O1	−0.5 (16)
O20—P1—O21—Mo10	−64.9 (3)	C30—N9—C28—O1	176.3 (11)

### Hydrogen-bond geometry (Å, º)

**Table:**

*D*—H···*A*	*D*—H	H···*A*	*D*···*A*	*D*—H···*A*
C3—H4···O31^i^	0.96	2.72	3.289 (11)	118
C4—H7···O36^ii^	0.96	2.61	3.476 (12)	150
C7—H13···O7	0.96	2.65	3.473 (12)	145
C8—H15···O28^ii^	0.96	2.51	3.316 (13)	141
C8—H16···O5	0.96	2.58	3.424 (11)	147
C10—H18···O28^ii^	0.93	2.62	3.516 (10)	162
C15—H29···O16^i^	0.96	2.55	3.299 (12)	135
C15—H27···O32^iii^	0.96	2.52	3.440 (10)	160
C15—H28···O52^iv^	0.96	2.29	3.174 (10)	153
C16—H31···O6^iii^	0.96	2.63	3.541 (11)	159
C22—H41···O55	0.96	2.53	3.280 (12)	135
C22—H42···O11^iv^	0.96	2.45	3.305 (11)	149
C22—H43···O45	0.96	2.61	3.567 (13)	172
C23—H44···O17^v^	0.96	2.52	3.443 (11)	161
C25—H55···O24	0.93	2.53	3.337 (11)	145
C25—H55···O44	0.93	2.58	3.091 (10)	115
C28—H56···O36^v^	0.93	2.62	3.476 (11)	153
C30—H60···O36^v^	0.96	2.58	3.458 (14)	152

Symmetry codes: (i) *x*+1/2, −*y*+1/2, *z*−1; (ii) −*x*+1, −*y*+1, *z*−1/2; (iii) *x*, *y*, *z*−1; (iv) −*x*+1, −*y*, *z*−1/2; (v) *x*+1/2, −*y*+1/2, *z*.
